# Supplementary figures and images for: Effects of Feeding Corn Distillers Dried Grains with Solubles on Muscle Quality Traits and Lipidomics Profiling of Finishing Pigs
Source: Animals (Basel). 2023 Dec 14;13(24):3848. doi: 10.3390/ani13243848 (PMC10741057; doi:10.3390/ani13243848)

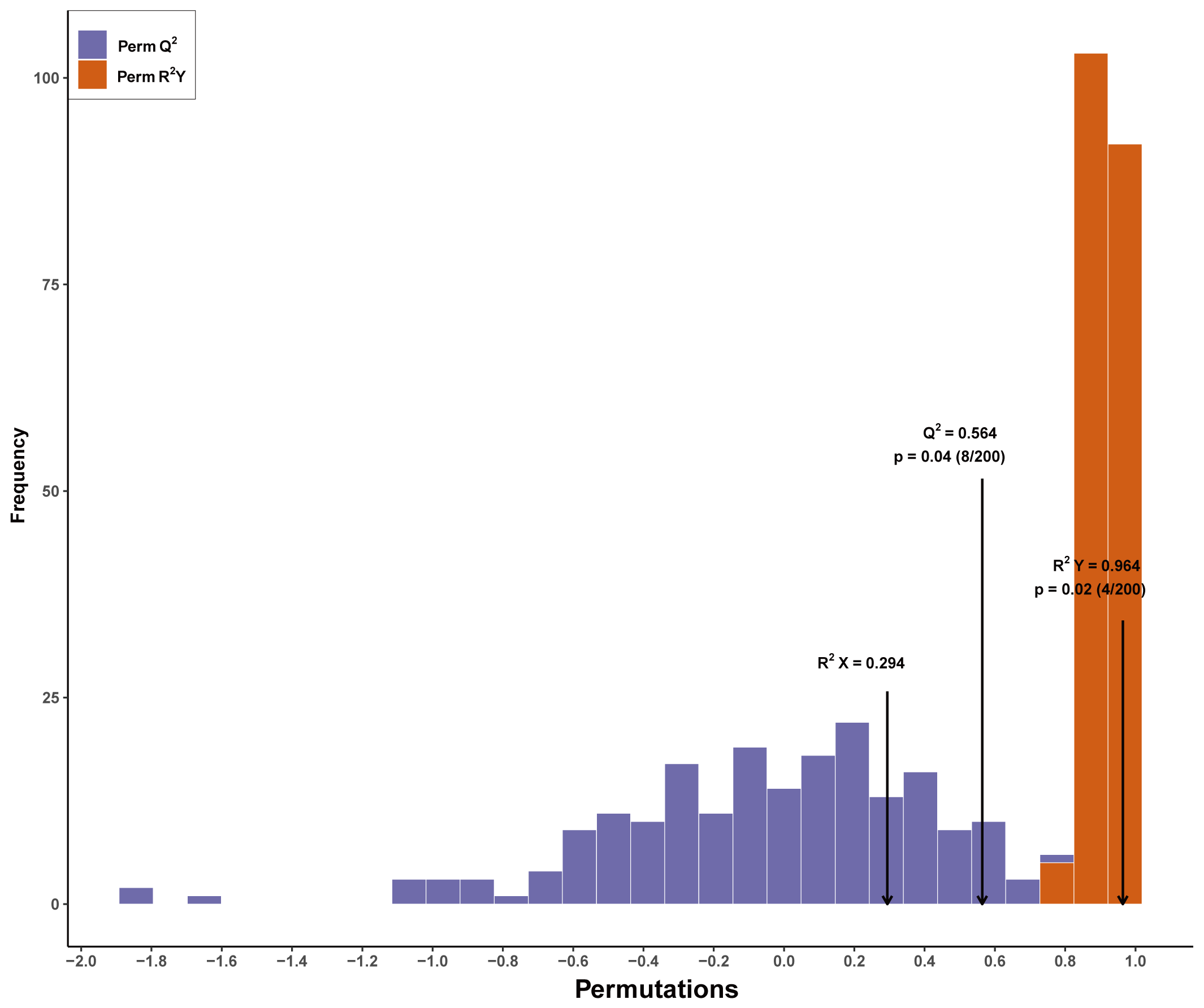

Supplement: Supplementary file 1 [file animals-13-03848-s001.zip › Supplementary Figure 1.tif]
